# Explaining pretrained language models' understanding of linguistic structures using construction grammar

**DOI:** 10.3389/frai.2023.1225791

**Published:** 2023-10-12

**Authors:** Leonie Weissweiler, Valentin Hofmann, Abdullatif Köksal, Hinrich Schütze

**Affiliations:** ^1^Center for Information and Language Processing, LMU Munich, Munich, Germany; ^2^Munich Center for Machine Learning, Munich, Germany; ^3^Faculty of Linguistics, University of Oxford, Oxford, United Kingdom

**Keywords:** NLP, probing, construction grammar, computational linguistics, large language models

## Abstract

Construction Grammar (CxG) is a paradigm from cognitive linguistics emphasizing the connection between syntax and semantics. Rather than rules that operate on lexical items, it posits *constructions* as the central building blocks of language, i.e., linguistic units of different granularity that combine syntax and semantics. As a first step toward assessing the compatibility of CxG with the syntactic and semantic knowledge demonstrated by state-of-the-art pretrained language models (PLMs), we present an investigation of their capability to classify and understand one of the most commonly studied constructions, the English comparative correlative (CC). We conduct experiments examining the classification accuracy of a syntactic probe on the one hand and the models' behavior in a semantic application task on the other, with BERT, RoBERTa, and DeBERTa as the example PLMs. Our results show that all three investigated PLMs, as well as OPT, are able to recognize the structure of the CC but fail to use its meaning. While human-like performance of PLMs on many NLP tasks has been alleged, this indicates that PLMs still suffer from substantial shortcomings in central domains of linguistic knowledge.

## 1. Introduction

The sentence “The better your syntax, the better your semantics.” contains a construction called the English comparative correlative (CC; Fillmore, 1986). Paraphrased, it could be read as “If your syntax is better, your semantics will also be better.” Humans reading this sentence are capable of doing two things: (i) *recognizing* that two instances of “the” followed by an adjective/adverb in the comparative as well as a phrase of the given structure (i.e., the syntax of the CC) express a specific meaning (i.e., the semantics of the CC); (ii) *understanding* the semantic meaning conveyed by the CC, i.e., understanding that in a sentence of the given structure, the second half is somehow correlated with the first.

In this paper, we ask the following question: are pretrained language models (PLMs) able to achieve these two steps? This question is important for two reasons. Firstly, we hope that recognizing the CC and understanding its meaning is challenging for PLMs, helping to set the research agenda for further improvements. Secondly, the CC is one of the most commonly studied constructions in construction grammar (CxG), a usage-based syntax paradigm from cognitive linguistics, thus providing an interesting alternative to the currently prevailing practice of analysing the syntactic capabilities of PLMs with theories from generative grammar (e.g., Marvin and Linzen, 2018).

We divide our investigation into two parts. In the first part, we examine the CC's syntactic properties and how they are represented by PLMs, with the objective to determine whether PLMs can *recognize* an instance of the CC. More specifically, we construct two syntactic probes with different properties: one is inspired by recent probing methodology (e.g., Belinkov et al., 2017; Conneau et al., 2018) and draws upon minimal pairs to quantify the amount of information contained in each PLM layer; for the other one, we write a context-free grammar (CFG) to construct approximate minimal pairs in which only the word order determines if the sentences are an instance of the CC or not. We find that starting from the third layer, all investigated PLMs are able to distinguish positive from negative instances of the CC. However, this method only covers one specific subtype of comparative sentences. To cover the full diversity of instances, we conduct an additional experiment for which we collect and manually label sentences from C4 (Raffel et al., 2020) that resemble instances of the CC, resulting in a diverse set of sentences that either are instances of the CC or resemble them closely *without* being instances of the CC. Applying the same methodology to this set of sentences, we observe that all examined PLMs are still able to separate the examples very well.

In the second part of the paper, we aim to determine if the PLMs are able to *understand* the meaning of the CC. We generate test scenarios in which a statement containing the CC is given to the PLMs, which they then have to apply in a zero-shot manner. As this way of testing PLMs is prone to a variety of biases, we introduce several mitigating methods in order to determine the full capability of the PLMs. We find that neither the masked language models nor the autoregressive models that we investigated performed above chance level on this task.

We make three main contributions:

– We present the first comprehensive study examining how well PLMs can recognize and understand a CxG construction, specifically the English comparative correlative.
– We develop a way of testing the PLMs' recognition of the CC that overcomes the challenge of probing for linguistic phenomena not lending themselves to minimal pairs.
– We adapt methods from zero-shot prompting and calibration to develop a way of testing PLMs for their understanding of the CC.

## 2. Construction grammar and natural language processing

### 2.1. Construction grammar

A core assumption of generative grammar (Chomsky, 1988), which can be already found in Bloomfieldian structural linguistics (Bloomfield, 1933), is a strict separation of lexicon and grammar: grammar is conceptualized as a set of compositional and general rules that operate on a list of arbitrary and specific lexical items in generating syntactically well-formed sentences. This dichotomous view was increasingly questioned in the 1980s when several studies drew attention to the fact that linguistic units larger than lexical items (e.g., idioms) can also possess non-compositional meanings (Lakoff, 1987; Langacker, 1987; Fillmore et al., 1988; Fillmore, 1989). For instance, it is not clear how the effect of the words “let alone” (as in “she doesn't eat fish, let alone meat”) on both the syntax and the semantics of the rest of the sentence could be inferred from general syntactic rules (Fillmore et al., 1988). This insight about the ubiquity of stored form-meaning pairings in language is adopted as the central tenet of grammatical theory by Construction Grammar (CxG; see Hoffmann and Trousdale, 2013 for a comprehensive overview). Rather than a system divided into non-overlapping syntactic rules and lexical items, CxG views language as a structured system of constructions with varying granularities that encapsulate syntactic and semantic components as single linguistic signs—ranging from individual morphemes up to phrasal elements and fixed expressions (Goldberg A., 1995; Kay and Fillmore, 1999). In this framework, syntactic rules can be seen as emergent abstractions over similar stored constructions (Goldberg, 2003, 2006). A different set of stored constructions can result in different abstractions and thus different syntactic rules, which allows CxG to naturally accommodate for the dynamic nature of grammar as evidenced, for instance, by inter-speaker variability and linguistic change (Hilpert, 2006).

### 2.2. Why construction grammar for NLP?

There has recently been growing interest in developing probing approaches for PLMs based on CxG. We see these approaches as coming from two different motivational standpoints, summarized below.

#### 2.2.1. Constructions are essential for language modeling

According to CxG, meaning is encoded in abstract constellations of linguistic units of different sizes. Examples of these can be found in Table 1. This means that LMs, which the field of NLP is trying to develop to achieve human language competency, must also be able to assign meaning to these units to be full LMs. Their ability to assign meaning to words, or more specifically to subword units which are sometimes closer to morphemes than to words, has been shown at length (Reif et al., 2019; Wiedemann et al., 2019; Schwartz et al., 2022). The question therefore remains: are PLMs able to retrieve and use meanings associated with patterns involving multiple tokens? We do not take this to only mean contiguous, fixed expressions, but much more importantly, non-contiguous patterns with slots that have varying constraints placed on them. To imitate and match human language behavior, models of human language need to learn how to recognize these patterns, retrieve their meaning, apply this meaning to the context, and use them when producing language. Simply put, there is no way around learning constructions if LMs are to advance. In addition, we believe that it is an independently interesting question whether existing PLMs pick up on these abstract patterns using the current architectures and training setups, and if not, which change in architecture would be necessary to facilitate this.

**Table 1:** Standard examples of constructions at various levels, adapted from Goldberg (2013).

**Construction name**	**Construction template**	**Examples**
Word		Banana
Word (partially filled)	pre-N, V-ing	Pretransition, Working
Idiom (filled)		Give the devil his due
Idiom (partially filled)	Jog < someone's> memory	She jogged his memory
Idiom (minimally filled)	The X-er the Y-er	The more I think about it, the less I know
Ditransitive construction (unfilled)	Subj V Obj1 Obj2	He baked her a muffin
Passive (unfilled)	Subj aux VPpp (PP by)	The armadillo was hit by a car

#### 2.2.2. Importance in downstream tasks

Regardless of more fundamental questions about the long-term goals of LMs, we also firmly believe that probing for CxG is relevant for analysing the challenges that face applied NLP, as evaluated on downstream tasks, at this point in time. Discussion is increasingly focusing on diagnosing the specific scenarios that are challenging for current models. Srivastava et al. (2023) propose test suites that are designed to challenge LMs, and many of them are designed by looking for “patterns” with a non-obvious, non-literal meaning that is more than the sum of the involved words. One example of such a failure can be found in Table 2, where we provide the DeepL^1^ translations for the famous instance of the caused-motion construction (Goldberg A. E., 1995, CMC): “She sneezed the foam off her cappuccino,” where the unusual factor is that *sneeze* does not usually take a patient argument or cause a motion. For translation, this means that it either has to use the corresponding CMC in the target language, which might be quite different in form from the English CMC, or paraphrase in a way that conveys all meaning facets. For the languages we tested, DeepL did not achieve this: the resulting sentence sounds more like the foam was sneezed onto the cappuccino, or is ambiguous between this and the correct translation. Interestingly, for Russian, the motion is conveyed in the translation, but not the fact that it is caused by a sneeze.

**Table 2:** Translated back to English by humans, they all mean “She sneezed her cappuccino's foam,” which does not correctly convey the resultative meaning component, i.e., that the foam is removed from the cappuccino by the sneeze (as opposed to put there).

**Lang**	**Reference translation**	**DeepL translation**
German	Sie nieste den Schaum von ihrem Cappuccino runter.	Sie nieste den Schaum von ihrem Cappuccino.
Italian	Lei ha starnutito via la schiuma dal suo cappuccino.	Starnutì la schiuma del suo cappuccino.
Turkish	Cappuccino'sunun köpüğünü hapşırdı.	Hapşırarak cappuccino'sunun köpüğünü uçurdu.

Targeted adversarial test suites like this translation example can be a useful resource to evaluate how well LMs perform on constructions, but more crucially, CxG theory and probing methods will inform the design of better and more systematic test suites, which in turn will be used to improve LMs.

#### 2.2.3. Diversity in linguistics for NLP

Discussions about PLMs as models of human language processing have recently gained popularity. One forum for such discussions is the Neural Nets for Cognition Discussion Group at CogSci2022^2^. The work is still very tentative, and most people agree that LMs are not ready to be used as models of human language processing. However, the discussion about whether LMs are ready to be used as cognitive models is dominated by results of probing studies based on Generative Grammar (GG), or more specifically Transformational Grammar. This means that GG is being used as the gold standard against which the cognitive plausibility of LMs is evaluated. Studies using GG assume a direct relationship between the models' performance on probing tasks and their linguistic competency. Increased performance on GG probing tasks is seen as a sign it is becoming more reasonable to use LMs as cognitive models. Another linguistic reason for theoretical diversity is that if we could show that LMs conform better to CxG rather than GG, this might open up interesting discussions if they ever start being used as cognitive models.

## 3. The English comparative correlative

The English comparative correlative (CC) is one of the most commonly studied constructions in linguistics, for several reasons. Firstly, it constitutes a clear example of a linguistic phenomenon that is challenging to explain in the framework of generative grammar (Culicover and Jackendoff, 1999; Abeillé and Borsley, 2008), even though there have been approaches following that school of thought (Den Dikken, 2005; Iwasaki and Radford, 2009). Secondly, it exhibits a range of interesting syntactic and semantic features, as detailed below. These reasons, we believe, also make the CC an ideal testbed for a first study attempting to extend the current trend of syntax probing for rules by developing methods for probing according to CxG.

The CC can take many different forms, some of which are exemplified here:

1. The more, the merrier.
2. The longer the bake, the browner the color.
3. The more she practiced, the better she became.

Semantically, the CC consists of two clauses, where the second clause can be seen as the dependent variable for the independent variable specified in the first one (Goldberg, 2003). It can be seen on the one hand as a statement of a general cause-and-effect relationship, as in a general conditional statement [e.g., (2) could be paraphrased as “If the bake is longer, the color will be more brown”], and on the other as a temporal development in a comparative sentence [paraphrasing (3) as “She became better over time, and she practiced more over time”]. Usage of the CC typically implies both readings at the same time. Syntactically, the CC is characterized in both clauses by an instance of “the” followed by an adverb or an adjective in the comparative, either with “-er” for some adjectives and adverbs, or with “more” for others, or special forms like “better.” Special features of the comparative sentences following this are the optional omission of the future “will” and of “be,” as in (1). Crucially, “the” in this construction does not function as a determiner of noun phrases (Goldberg, 2003); rather, it has a function specific to the CC and has variously been called a “degree word” (Den Dikken, 2005) or “fixed material” (Hoffmann et al., 2019).

## 4. Related work

### 4.1. Construction grammar probing

#### 4.1.1. CxGBERT

Tayyar Madabushi et al. (2020) investigate how well BERT (Devlin et al., 2019) can classify whether two sentences contain instances of the same construction. Their list of constructions is extracted with a modified version of Dunn (2017)'s algorithm: they induce a CxG in an unsupervised fashion over a corpus, using statistical association measures. Their list of constructions is taken directly from Dunn (2017), and they find their instances by searching for those constructions' occurrences in WikiText data. This makes the constructions possibly problematic, since they have not been verified by a linguist, which could make the conclusions drawn later from the results about BERT's handling of constructions hard to generalize from.

The key probing question of this paper is: Do two sentences contain the same construction? This does not necessarily need to be the most salient or overarching construction of the sentence, so many sentences will contain more than one instance of a construction. Crucially, the paper does not follow a direct probing approach, but rather finetunes or even trains BERT on targeted construction data, to then measure the impact on CoLA. They find that on average, models trained on sentences that were sorted into documents based on their constructions do not reliably perform better than those trained on original, unsorted data. However, they additionally test BERT Base with no additional pre-training on the task of predicting whether two sentences contain instances of the same construction, measuring accuracies of about 85% after 500 training examples for the probe. These results vary wildly depending on the frequency of the construction, which might relate back to the questionable quality of the automatically identified list of constructions.

#### 4.1.2. Neural reality of argument structure constructions

Li et al. (2022) probe for LMs' handling of four argument structure constructions: ditransitive, resultative, caused-motion, and removal. Specifically, they attempt to adapt the findings of Bencini and Goldberg (2000), who used a sentence sorting task to determine whether human participants perceive the argument structure or the verb as the main factor in the overall sentence meaning. The paper aims to recreate this experiment for MiniBERTa (Warstadt et al., 2020b) and RoBERTa (Liu et al., 2019), by generating sentences artificially and using agglomerative clustering on the sentence embeddings. They find that, similarly to the human data, which is sorted by the English proficiency of the participants, PLMs increasingly prefer sorting by construction as their training data size increases. Crucially, the sentences constructed for testing had no lexical overlap, such that this sorting preference must be due to an underlying recognition of a shared pattern between sentences with the same argument structure. They then conduct a second experiment, in which they insert random verbs, which are incompatible with one of the constructions, and then measure the Euclidean distance between this verb's contextual embedding and that of a verb that is prototypical for the corresponding construction. The probing idea here is that if construction information is picked up by the model, the contextual embedding of the verb should acquire some constructional meaning, which would bring it closer to the corresponding prototypical verb meaning than to the others. They indeed find that this effect is significant, for both high and low frequency verbs.

#### 4.1.3. CxLM

Tseng et al. (2022) study LM predictions for the slots of various degrees of openness for a corpus of Chinese constructions. Their original data comes from a knowledge database of Mandarin Chinese constructions (Zhan, 2017), which they filter so that only constructions with a fixed repetitive element remain, which are easier to find automatically in a corpus. They filter this list down further to constructions which are rated as commonly occurring by annotators, and retrieve instances from a POS-tagged Taiwanese bulletin board corpus. They binarize the openness of a given slot in a construction and mark each word in a construction as either constant or variable. The key probing idea is then to examine the conditional probabilities that a model outputs for each type of slot, with the expectation that the prediction of variable slot words will be more difficult than that of constant ones, providing that the model has acquired some constructional knowledge. They find that this effect is significant for two different Chinese BERT-based models, as negative log-likelihoods are indeed significantly higher when predicting variable slots compared to constant ones. Interestingly, the negative log-likelihood resulting from masking the entire construction lies in the middle of the two extremes. They further evaluate a BERT-based model which is finetuned on just predicting the variable slots of the dataset they compiled and find, unsurprisingly, that this improves accuracy greatly.

#### 4.1.4. A discerning several thousand judgments

Mahowald (2023) focuses on the English Article + Adjective + Numeral + Noun (AANN) construction, e.g. “The president has had a terrible 5 weeks” and GPT-3's recognition of its particular semantic and syntactic constraints. He designs a few-shot prompt for grammatical acceptability using the CoLA corpus of linguistic acceptability (Warstadt et al., 2019). As probing data, he artificially constructs several variants of the AANN construction to test for GPT-3's understanding of its properties. Its output on the linguistic acceptability task is also contrasted with human ratings sourced from Mechanical Turk. The probing concept exploits that the AANN construction has several properties that seem to violate a number of rules: “a” is not marking a singular here, as the noun is plural. Also, the order of the number and the adjective is reversed, and in some cases, verb agreement rules must be suspended. There are also interesting constraints on the construction itself: for example, some adjectives, such as color words, are not acceptable. Furthermore, qualitative adjectives must appear before quantitative ones. Overall, GPT-3 judgments match the direction of the human ones across a variety of conditions, except on the question of quantitative vs qualitative adjectives, where humans showed no preference, and GPT-3 had a slightly preference against the one described in the literature. This shows that the model understood the syntactic structure of the AANN construction to the point where it can override more global “rules” about word order, but makes no statement about its understanding of the meaning.

### 4.2. NLP and construction grammar

Other computational studies about CxG have either focused on automatically annotating constructions (Dunietz et al., 2017) or on the creation and evaluation of automatically built lists of constructions (Marques and Beuls, 2016; Dunn, 2019).

### 4.3. General probing

Our work also bears some similarity to recent work in generative grammar-based syntax probing of large PLMs in that we approximate the minimal pairs-based probing framework similar to Wei et al. (2021), Marvin and Linzen (2018), or Goldberg (2019). However, as we are concerned with different phenomena and investigating them from a different theoretical standpoint, the syntactic half of our work clearly differs.

The semantic half of our study is closest to recent work on designing challenging test cases for models such as Ribeiro et al. (2020), who design some edge cases for which most PLMs fail. Despite the different motivation, the outcome is very similar to a list of some particularly challenging constructions.

## 5. Syntax

Our investigation of PLMs' knowledge of the CC is split into two parts. First, we probe for the PLMs' knowledge of the syntactic aspects of the CC, to determine if they recognize its structure. Then we devise a test of their understanding of its semantic aspects by investigating their ability to apply, in a given context, information conveyed by a CC.

### 5.1. Probing methods

As the first half of our analysis of PLMs' knowledge of the CC, we investigate its syntactic aspects. Translated into probing questions, this means that we ask: can a PLM recognize an instance of the CC? Can it distinguish instances of the CC from similar-looking non-instances? Is it able to go beyond the simple recognition of its fixed parts (“The COMP-ADJ/ADV, the ...”) and group all ways of completing the sentences that are instances of the CC separately from all those that are not? And to frame all of these questions in a syntactic probing framework: will we be able to recover, using a logistic regression as the probe, this distinguishing information from a PLM's embeddings?

The established way of testing a PLM for its syntactic knowledge has in recent years become minimal pairs (e.g., Warstadt et al., 2020a; Demszky et al., 2021). This would mean pairs of sentences which are indistinguishable except for the fact that one of them is an instance of the CC and the other is not, allowing us to perfectly separate a model's knowledge of the CC from other confounding factors. While this is indeed possible for simpler syntactic phenomena such as verb-noun number agreement, there is no obvious way to construct minimal pairs for the CC. We therefore construct minimal pairs in two ways: one with artificial data based on a context-free grammar (CFG), and one with sentences extracted from C4.

#### 5.1.1. Synthetic data

In order to find a pair of sentences that is as close as possible to a minimal pair, we devise a way to modify the words following “The X-er” such that the sentence is no longer an instance of the construction. The pattern for a positive instance is “The ADV-er the NUM NOUN VERB,” e.g., “The harder the two cats fight.” To create a negative instance, we reorder the pattern to “The ADJ-er NUM VERB the NOUN,” e.g., “The harder two fight the cats.” The change in role of the numeral from the dependent of a head to a head itself, made possible by choosing a verb that can be either transitive or intransitive, as well as the change from an adverb to an adjective, allows us to construct a negative instance that uses the same words as the positive one, but in a different order.^3^ In order to generate a large number of instances, we collect two sets each of adverbs, numerals, nouns, and verbs that are mutually exclusive between training and test sets. To investigate if the model is confused by additional content in the sentences, we write an CFG to insert phrases before the start of the first half, in between the two halves, and after the second half of the CC. We show the rules making up the CFG in Algorithms 1, 2.

**Algorithm 1:** Context-free grammar for artificial data creation training set.

S → SPOS | SNEG
SPOS → POS1 PUNCT POS2 ‘.' | POS1 INSERT PUNCT POS2 ‘.'
SNEG → NEG1 PUNCT NEG2 ‘.' | NEG1 INSERT PUNCT NEG2 ‘.'
PUNCT → ‘,' | ‘;' | ϵ
CORE_POS → ADV_I ‘the' NUM NOUN VERB
CORE_NEG → ADV_I NUM VERB ‘the' NOUN
POS_UPPER → ‘0 The' CORE_POS
POS_LOWER → ‘0 the' CORE_POS
NEG_UPPER → ‘0 The' CORE_NEG
NEG_LOWER → ‘0 the' CORE_NEG
POS1 → POS_UPPER | POS_UPPER ADD | START POS_LOWER | START POS_LOWER ADD
POS2 → POS_LOWER | POS_LOWER ADD
NEG1 → NEG_UPPER | NEG_UPPER ADD | START NEG_LOWER | START NEG_LOWER ADD
NEG2 → NEG_LOWER | NEG_LOWER ADD
INSERT → INSERT1 | INSERT2
INSERT2 → ADDITION BETWEEN_ADD_AND_SENT SENT
PRON → ‘we' | ‘they'
ADDITION → ‘, and by the way,' | ‘, and I want to add that' | ‘, and' PRON ‘just want to say that' | ‘, and then' PRON ‘said that' | ‘, and then' PRON ‘said that'
SAY → ‘say' | ‘think' | ‘mean' | ‘believe'
BETWEEN_ADD_AND_SENT → PRON SAY ‘that' | PRON SAY ‘that' | PRON SAY ‘that' | PRON SAY ‘that'
LOC_SENT → PRON ‘said this in' LOC ‘too'
LOC → CITY ‘and' LOC | CITY
CITY → ‘Munich' | ‘Washington' | ‘Cologne' | ‘Prague' | ‘Istanbul'
SENT → ‘this also holds in other cases' | ‘this is not always true' | ‘this is always true' | ‘this has only recently been the case' | ‘this has not always been the case' | ‘this has always been the case'
INSERT1 → ‘without stopping' | ‘without a break' | ‘without a pause' | ‘uninterrupted'
START → ‘Nowadays, ' | ‘Nowadays' | ‘Therefore, ' | ‘Therefore' | ‘We can' CANWORD ‘that' | ‘It is' KNOWNWORD ‘that' | ‘It follows that' | ‘Sometimes'
START → Sometimes,' | It was recently announced that' | People have told me that' | I recently read in a really interesting book that' | I have recently read in an established, well-known newspaper that' | It was reported in a special segment on TV today that'
CANWORD → say' | surmise' | accept' | state'
KNOWNWORD → clear' | known' | accepted' | obvious'
ADD → TEMP | UNDER1 | TEMP UNDER1 | UNDER1 TEMP
ADV_I → ADV | ADV and' ADV
TEMP → TEMP1 TEMP2
TEMP1 → before' | after' | during'
TEMP2 → the morning' | the afternoon' | the night'
UNDER1 → under the' UNDER2
UNDER2 → bed' | roof' | sun'
VERB → push' | attack' | chase' | beat' | believe' | boil' | box' | burn' | call' | date'
NOUN → lions' | pandas' | camels' | pigs' | horses' | sheep' | chickens' | foxes' | cows' | deer'
ADV → worse' | earlier' | slower' | deeper' | bigger' | smaller' | flatter' | weaker' | stronger' | louder'
NUM → twelve' | thirteen' | fourteen' | fifteen' | sixteen' | seventeen' | eighteen' | nineteen' | twenty' | ‘twenty-one'

**Algorithm 2:** Context-free grammar for artificial data creation test set.

S → SPOS | SNEG
SPOS → POS1 PUNCT POS2 '.' | POS1 INSERT PUNCT POS2 '.'
SNEG → NEG1 PUNCT NEG2 '.' | NEG1 INSERT PUNCT NEG2 '.'
PUNCT → ',' | ';' | ”
CORE_POS → ADV_I 'the' NUM NOUNVERB
CORE_NEG → ADV_I NUM VERB 'the' NOUN
POS_UPPER → '0 The' CORE_POS
POS_LOWER → '0 the' CORE_POS
NEG_UPPER → '0 The' CORE_NEG
NEG_LOWER → '0 the' CORE_NEG
POS1 → POS_UPPER | POS_UPPER ADD | START POS_LOWER | START POS_LOWER ADD
POS2 → POS_LOWER | POS_LOWER ADD
NEG1 → NEG_UPPER | NEG_UPPER ADD | START NEG_LOWER | START NEG_LOWER ADD
NEG2 → NEG_LOWER | NEG_LOWER ADD
INSERT → INSERT1 | INSERT2
INSERT2 → ADDITION BETWEEN_ADD_AND_SENT SENT
PRON → 'I' | 'you'
ADDITION → ', and by the way ,' | ', and I want to add that' | ', and' PRON 'just want to say that' | ', and then' PRON 'said that' | ', and then' PRON 'said that'
SAY → 'say' | 'think' | 'mean' | 'believe'
BETWEEN_ADD_AND_SENT → PRON SAY 'that' | PRON SAY 'that' | PRON SAY 'that' | PRON SAY 'that'
LOC_SENT → PRON 'said this in' LOC 'too'
LOC → CITY 'and' LOC | CITY
CITY → 'London' | 'New York' | 'Berlin' | 'Madrid' | 'Paris'
SENT → 'this also holds in other cases' | 'this is not always true' | 'this is always true' | 'this has only recently been the case' | 'this has not always been the case' | 'this has always been the case'
INSERT1 → 'without stopping' | 'without a break' | 'without a pause' | 'uninterrupted' |
START → 'Nowadays ,' | 'Nowadays' | 'Therefore ,' | 'Therefore' | 'We can' CANWORD 'that' | 'It is' KNOWNWORD 'that' | 'It follows that' | 'Sometimes' | 'Sometimes ,' | 'It was recently announced that' | 'People have told me that' | 'I recently read in a really interesting book that' | 'I have recently read in an established , well-known newspaper that' | 'It was reported in a special segment on TV today that'
CANWORD → 'say' | 'surmise'
KNOWNWORD → 'clear' | 'known'
ADD → TEMP | UNDER1 | TEMP UNDER1 | UNDER1 TEMP
ADV_I → ADV | ADV 'and' ADV
TEMP → TEMP1 TEMP2
TEMP1 → 'before' | 'after' | 'during'
TEMP2 → 'the day' | 'the night' | 'the evening'
UNDER1 → 'under the' UNDER2
UNDER2 → 'bridge' | 'stairs' | 'tree'
VERB → 'slam' | 'break' | 'bleed' | 'shake' | 'smash' | 'throw' | 'strike' | 'shoot' | 'swallow' | 'choke'
NOUN → 'cats' | 'dogs' | 'girls' | 'boys' | 'men' | 'women' | 'people' | 'humans' | 'mice' | 'alligators'
ADV → 'faster' | 'quicker' | 'harder' | 'higher' | 'later' | 'longer' | 'shorter' | 'lower' | 'wider' | 'better'
NUM → 'two' | 'three' | 'four' | 'five' | 'six' | 'seven' | 'eight' | 'nine' | 'ten' | 'eleven'

While this setup is rigorous in the sense that positive and negative sentences are exactly matched, it comes with the drawback of only considering one type of CC. To be able to conduct a more comprehensive investigation, we adopt a complementary approach and turn to pairs extracted from C4. We show examples of training and test data in Table 3. These cover a broad range of CC patterns, albeit without meeting the criterion that positive and negative samples are exactly matched.

**Table 3:** Examples of data for the syntactic probe.

**Sentence**	**Label**	**Source**
“The higher up the nicer!”	Positive	Corpus
She thinks the more water she drinks the better her skin looks.	Positive	Corpus
Subtract the smaller from the larger.	Negative	Corpus
The way the older guys help out the younger guys is fantastic.	Negative	Corpus
Nowadays, the bigger the 18 sheep date, the louder and bigger the 12 horses beat under the sun.	Positive	Artificial train
The flatter the 14 lions push, the deeper and smaller the 16 deer burn under the roof.	Positive	Artificial train
Sometimes, the worse and earlier 17 believe the deer, and we just want to say that they mean that this has always been the case, the flatter 21 attack the foxes before the afternoon under the roof.	Negative	Artificial train
Nowadays, the smaller 16 box the camels, and by the way, they mean that this is always true; the weaker 13 date the cows.	Negative	Artificial train
The harder and longer the three cats throw, the harder and shorter the 10 dogs shake.	Positive	Artificial test
I have recently read in an established, well-known newspaper that the later the ten mice strike; the later and better the seven men smash under the tree during the night.	Positive	Artificial test
The higher nine strike the women without a pause the shorter 10 choke the girls.	Negative	Artificial test
We can say that the longer and faster four strike the men under the stairs before the evening, the harder four throw the dogs after the day under the bridge.	Negative	Artificial test

#### 5.1.2. Corpus-based minimal pairs

While accepting that positive and negative instances extracted from a corpus will automatically not be minimal and therefore contain some lexical overlap and context cues, we attempt to regularize our retrieved instances as far as possible. To form a first candidate set, we POS tag C4 using spaCy (Honnibal and Montani, 2018) and extract all sentences that follow the pattern “The” (DET) followed by either “more” and an adjective or adverb, or an adjective or adverb ending in “-er,” and at any point later in the sentence again the same pattern. We discard examples with adverbs or adjectives that were falsely labeled as comparative, such as “other.” We then group these sentences by their sequence of POS tags, and manually classify the sequences as either positive or negative instances. We observe that sentences sharing a POS tag pattern tend to be either all negative or all positive instances, allowing us to save annotation time by working at the POS tag pattern level instead of the sentence level. To make the final set as diverse as possible, we sort the patterns randomly and label as many as possible. In order to further reduce interfering factors in our probe, we separate the POS tag patterns between training and test sets. We give examples in Table 3.

Please note that due to the inherent difficulty of creating minimal pairs for this construction, while the two approaches are complementary, neither of them is perfect. While we think that our experimental setup (e.g., no surface patterns indicating positive/negative classes, clear distinction between training/test data) is designed well-enough, we would like to note that probing classifiers with logistic regression are not robust to such confound variables.

#### 5.1.3. The probe

For both datasets, we investigate the overall accuracy of our probe as well as the impact of several factors. The probe consists of training a simple logistic regression model on top of the mean-pooled sentence embeddings (Vulić et al., 2020). To quantify the impact of the length of the sentence, the start position of the construction, the position of its second half, and the distance between them, we construct four different subsets $D_{f}^{\mathrm{train}}$ and $D_{f}^{\mathrm{test}}$ from both the artificially constructed and the corpus-based dataset. For each subset, we sample sentences such that both the positive and the negative class is balanced across every value of the feature within a certain range of values. This ensures that the probes are unable to exploit correlations between a class and any of the above features. We create the dataset as follows

$$D_{f}=\underset{v\in f_{v}}{⋃}\underset{l^{*}\in L}{⋃}S\left(D,v,l^{*},n^{*}\right),$$

where *f* is the feature, *f*_*v*_ is the set of values for *f*, *L* = {*positive, negative*} are the labels, and *S* is a function that returns n^*^ elements from *D* that have value *v* and label l^*^.

To make this task more cognitively realistic, we aim to test if a model is able to generalize from shorter sentences, which contain relatively little additional information besides the parts relevant to the classification task, to those with greater potential interference due to more additional content that is not useful for classification. Thus, we restrict the training set to samples from the lowest quartile of each feature so that *f*_*v*_ becomes $\left[v_{f}^{\min},v_{f}^{\min}+\frac{1}{4}\left(v_{f}^{\max}-v_{f}^{\min}\right)\right]$ for $D_{f}^{\mathrm{train}}$ and $\left[v_{f}^{\min},v_{f}^{\max}\right]$ for $D_{f}^{\mathrm{test}}$. We report the test performance for every value of a given feature separately to recognize patterns. For the artificial syntax probing, we generate 1,000 data points for each value of each feature for each training and test for each subset associated with a feature. For the corpus syntax probing, we collect 9,710 positive and 533 negative sentences in total, from which we choose 10 training and five test sentences for each value of each feature in a similar manner. To improve comparability and make the experiment computationally feasible, we test the “large” size of each of our three models, using the Huggingface Transformers library (Wolf et al., 2019). Our logistic regression probes are implemented using Scikitlearn (Pedregosa et al., 2011).

### 5.2. Probing results

#### 5.2.1. Artificial data

**Figure 1:**
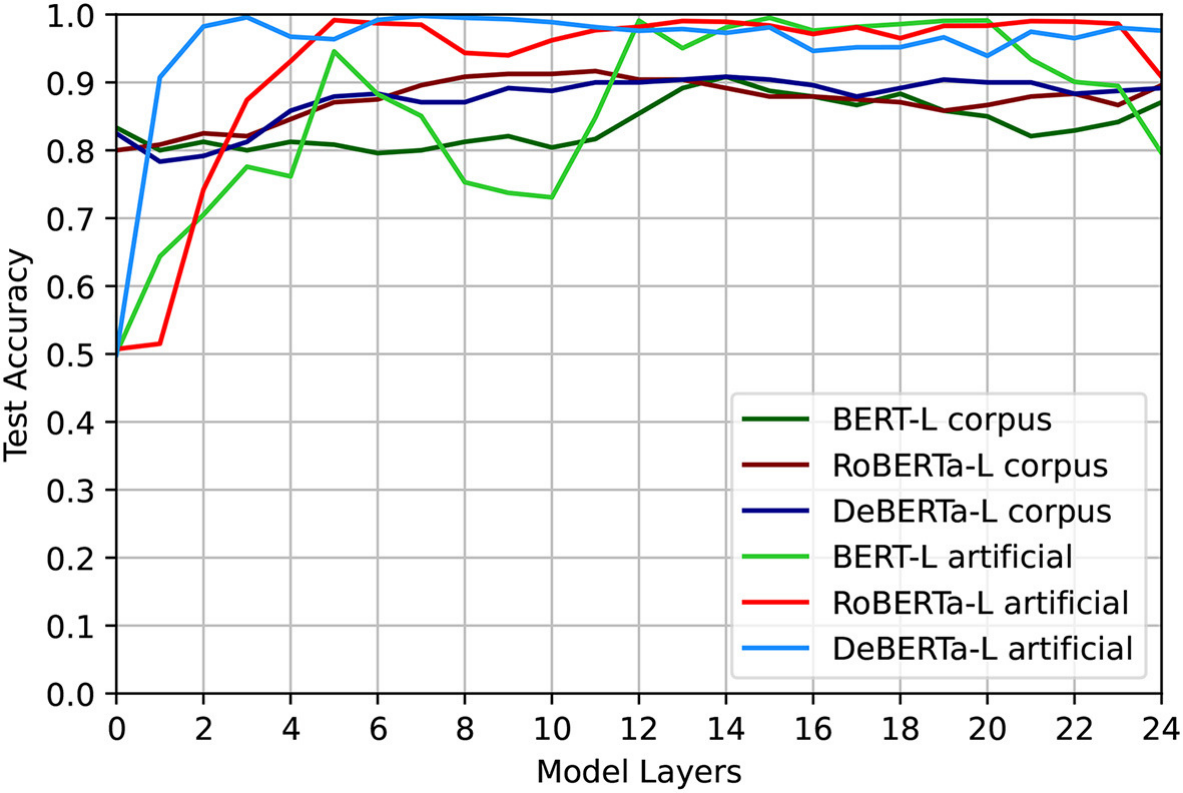
Overall accuracy per layer for *D*_length_. All shown models are the large model variants. The models can easily distinguish between positive and negative examples in at least some of their layers.

As shown in Figure 1, the results of our syntactic probe indicate that all models can easily distinguish between positive and negative examples in at least some of their layers, independently of any of the sentence properties that we have investigated. We report full results in Figures A1–A3 in the Appendix (Supplementary material). We find a clear trend that DeBERTa performs better than RoBERTa, which in turn performs better than BERT across the board. As DeBERTa's performance in all layers is nearly perfect, we are unable to observe patterns related to the length of the sentence, the start position of the CC, the start position of the second half of the CC, and the distance between them. By contrast, we observe interesting patterns for BERT and RoBERTa. For *D*_length_, and to a lesser degree *D*_distance_ (which correlates with it), we observe that at first, performance goes down with increased length as we would expect—the model struggles to generalize to longer sentences with more interference since it was only trained on short ones. However, this trend is reversed in the last few layers. We hypothesize this may be due to an increased focus on semantics in the last layers (Peters et al., 2018; Tenney et al., 2019), which could lead to interfering features particularly in shorter sentences.

#### 5.2.2. Corpus data

In contrast, the results of our probe on more natural data from C4 indicate two different trends: first, as the positive and negative instances are not identical on a bag-of-word level, performance is not uniformly at 50% (i.e., chance) level in the first layers, indicating that the model can exploit lexical cues to some degree. We observe a similar trend as with the artificial experiment, which showed that DeBERTa performs best and BERT worst. The corresponding graphs can be found in Figures A4–A6 in Supplementary material.

Generally, this additional corpus-based experiment validates our findings from the experiment with artificially generated data, as all models perform at 80% or better from the middle layers on, indicating that the models are able to classify instances of the construction even when they are very diverse and use unseen POS tag patterns.

Comparing the average accuracies on *D*_length_ for both data sources in Figure 1, we observe that all models perform better on artificial than on corpus data from the fifth layer on, with the notable exception of a dip in performance for BERT large around layer 10.

## 6. Semantics

### 6.1. Probing approach

For the second half of our investigation, we turn to semantics. In order to determine if a model has understood the meaning of the CC, i.e., if it has understood that in any sentence, “the COMP .... the COMP” implies a correlation between the two halves, we adopt a usage-based approach and ask: can the model, based on the meaning conveyed by the CC, draw a correct inference in a specific scenario? For this, we construct general test instances of the CC that consist of a desired update of the belief state of the model about the world, which we then expect it to be able to apply. More concretely, we generate sentences of the form “The ADJ1-er you are, the ADJ2-er you are.,” while picking adjectives at random. To this general statement, we then add a specific scenario with two random names: “NAME1 is ADJ1-er than NAME2.” and ask the model to draw an inference from it. We first construct a test scenario for this that works with masked language models and test BERT, RoBERTa and DeBERTa on it, and then modify the setup and move on to autoregressive models, specifically OPT (Zhang et al., 2022).

### 6.2. Experiments on masked language models

#### 6.2.1. Probing methods

In our experiments with masked language models, we now ask the models to draw an inference from the context by predicting a token at the masked position in the following sentence: “Therefore, NAME1 is [MASK] than NAME2.” If the model has understood the meaning conveyed by the CC and is able to use it in predicting the mask, we expect the probability of ADJ2 to be high.

To provide the model with an alternative, we add a second sentence, another instance of the CC, using the antonyms of the two adjectives. This sentence is carefully chosen to have no impact on the best filler for [MASK], but also for other reasons explained in Section 6.2.1.1. The full test context is shown in Table 5, S1. This enables us to compare the probability of ADJ2 for the mask token directly with a plausible alternative, ANT2. One of our test sentences might be “The stronger you are, the faster you are. The weaker you are, the slower you are. Terry is stronger than John. Therefore, Terry will be [MASK] than John,” where we compare the probabilities of “faster” and “slower.”

Note that success in our experiment does not necessarily indicate that the model has fully understood the meaning of the CC. The experiment can only provide a lower bound for the underlying understanding of any model. However, we believe that our task is not unreasonable for a masked language model in a zero-shot setting. It is comparable in difficulty and non-reliance on world knowledge to the NLU tasks presented in LAMBADA (Paperno et al., 2016), on which GPT-2 (117 M to 1.5 B parameters) has achieved high zero-shot accuracy (Radford et al., 2019, Table 4). While we investigate masked language models and not GPT-2, our largest models are comparable in size to the sizes of GPT-2 that were used (340 M for BERT_L_, 355 M for RoBERTa_L_, and 1.5 B parameters for DeBERTa-XXL_L_), and we believe that this part of our task is achievable to some degree.

**Table 4:** Overview of constructions investigated in CxG-specific probing literature, with examples.

**References**	**Language**	**Source**	**Construction**	**Example**
Tayyar Madabushi et al. (2020)	English	From automatically constructed list by Dunn (2017)	Personal Pronoun + didn't + V + how	We didn't know how or why.
Li et al. (2022)	English	Argument structure constructions according to Bencini and Goldberg (2000)	caused-motion	Bob cut the bread into the pan.
Tseng et al. (2022)	Chinese	From constructions list by Zhan (2017)	a + 到 + 爆, etc.	好吃到爆了! *It's so delicious!*
Weissweiler et al. (2022)	English	McCawley (1988)	Comparative correlative	The bigger, the better.
Mahowald (2023)	English	Jackendoff (1977)	Article + Adjective + Numeral + Noun	A lovely 5 days

##### 6.2.1.1. Biases

In this setup, we hypothesize several biases that models could exhibit and might cloud our assessment of its understanding of the CC, and devise a way to test their impact.

Firstly, we expect that models might prefer to repeat the adjective that is closest to the mask token. This has recently been documented for prompt-based experiments (Zhao et al., 2021). Here, this adjective is ANT2, the wrong answer. To test the influence this has on the prediction probabilities, we construct an alternative version of our test context in which we flip the first two sentences so that the correct answer is now more recent. The result can be found in Table 5, S2.

**Table 5:** Overview of the schemata of all test scenarios used for semantic probing for masked language models.

**No**.	**Purpose**	**Approach**	**Sentence schema**
S1	Base		The ADJ1-er you are, the ADJ2-er you are. The ANT1-er you are, the ANT2-er you are.
			NAME1 is ADJ1-er than NAME2. Therefore, NAME1 is [MASK] than NAME2.
S2	Bias test	Recency	The ANT1-er you are, the ANT2-er you are. The ADJ1-er you are, the ADJ2-er you are.
			NAME1 is ADJ1-er than NAME2. Therefore, NAME1 is [MASK] than NAME2.
S3		Vocabulary	The ADJ1-er you are, the ANT2-er you are. The ANT1-er you are, the ADJ2-er you are.
			NAME2 is ADJ1-er than NAME2. Therefore, NAME1 is [MASK] than NAME2.
S4		Name	The ADJ1-er you are, the ADJ2-er you are. The ANT1-er you are, the ANT2-er you are.
			NAME2 is ADJ1-er than NAME1. Therefore, NAME2 is [MASK] than NAME1.
S5	Calibration	Short	NAME1 is ADJ1-er than NAME2. Therefore, NAME1 is [MASK] than NAME2.
S6		Name	The ADJ1-er you are, the ADJ2-er you are. The ANT1-er you are, the ANT2-er you are.
			NAME1 is ADJ1-er than NAME2. Therefore, NAME3 is [MASK] than NAME4.
S7		Adjective	The ADJ1-er you are, the ADJ2-er you are. The ANT1-er you are, the ANT2-er you are.
			NAME1 is ADJ3-er than NAME2. Therefore, NAME1 is [MASK] than NAME2.

Secondly, we expect that models might assign higher probabilities to some adjectives, purely based on their frequency in the pretraining corpus, as for example observed by Holtzman et al. (2021). To test this, we construct a version of the test context in which ADJ2/ANT2 are swapped, which means that we can keep both the overall words the same as well as the position of the correct answer, while changing which adjective it is. The sentence is now S3 in Table 5. If there is a large difference between the prediction probabilities for the two different versions, that this means that a model's prediction is influenced by the lexical identity of the adjective in question.

Lastly, a model might have learned to associate adjectives with names in pretraining, so we construct a third version, in which we swap the names. This is S4 in Table 5. If any prior association between names and adjectives influences the prediction, we expect the scores between S4 and S1 to differ.

##### 6.2.1.2. Calibration

After quantifying the biases that may prevent us from seeing a model's true capability in understanding the CC, we aim to develop methods to mitigate it. We turn to calibration, which has recently been used in probing with few-shot examples by Zhao et al. (2021). The aim of calibration is to improve the performance of a model on a classification task, by first assessing the prior probability of a label (i.e., its probability if no context is given), and then dividing the probability predicted in the task context by this prior; this gives us the conditional probability of a label given the context, representing the true knowledge of the model about this task. In adapting calibration, we want to give a model every possible opportunity to do well so that we do not underestimate its underlying comprehension.

We therefore develop three different methods of removing the important information from the context in such a way that we can use the prediction probabilities of the two adjectives in these contexts for calibration. The simplest way of doing this is to remove both instances of the CC, resulting in S5 in Table 5. If we want to keep the CC in the context, the two options to remove any information are to replace either the names or the adjectives with new names/adjectives. We therefore construct two more instances for calibration: S6 and S7 in Table 5.

For each calibration method, we collect five examples with different adjectives or names. For a given base sample *S*_*b*_, we calculate *P*_*c*_, the calibrated predictions, as follows:

$$\begin{array}{l} P_{c}\left(a|S_{b}\right)=P\left(a|S_{b}\right)/\left[\overset{i=5}{\underset{i=1}{\sum }}\left(P\left(a|C_{i}\right)/5\right)\right] \end{array}$$

where *C*_*i*_ is the *i*-th example of a given calibration technique, *a* is the list of adjectives tested for the masked position, and the division is applied elementwise. We collect a list of 20 adjectives and their antonyms manually from the vocabulary of the RoBERTa tokenizer and 33 common names and generate 144,800 sentences from them. We test BERT (Devlin et al., 2019) in the sizes base and large, RoBERTa (Liu et al., 2019) in the sizes base and large, and DeBERTa (He et al., 2020) in the sizes base, large, xlarge, and xxlarge.

#### 6.2.2. Results

In Table 6, we report the accuracy for all examined models. Out of the three variations to test biases, we report accuracy only for the sentence testing the recency bias as we expect this bias to occur systematically across all sentences: if it is a large effect, it will always lead to the sentence where the correct answer is the more recent one being favored. To assess the influence of each bias beyond accuracy, we report as decision flip the percentage of sentences for which the decision (i.e., if the correct adjective had a higher probability than the incorrect one) was changed when considering the alternative sentence that was constructed to test for bias. We report full results in Table 7.

**Table 6:** Selected accuracies and results for the semantic probe.

	**Accuracy**		**Decision flip**		
	**S1**	**S2**	**S2**	**S3**	**S4**
BERT_B_	37.65	64.64	26.98	75.69	02.70
BERT_L_	36.85	67.21	30.44	73.31	02.32
RoBERTa_B_	61.60	52.84	09.91	76.18	02.76
RoBERTa_L_	55.71	68.00	14.33	79.47	04.33
DeBERTa_B_	49.72	49.80	00.91	99.66	01.07
DeBERTa_L_	50.88	51.40	07.04	94.83	02.23
DeBERTa_XL_	47.73	49.33	05.46	89.28	02.51
DeBERTa_XXL_	47.34	48.72	03.59	82.09	01.13

We report the average accuracy on the more difficult sentences in terms of recency bias (S1) and the easier ones (S2), as well as the percentage of decisions flipped by changing from the base S1 to the sentences testing for recency bias (S2), vocabulary bias (S3), and name bias (S4). RoBERTa and DeBERTa perform close to chance on S1 and S2 accuracy, indicating that they do not understand the meaning of CC. BERT's performance is strongly influenced by biases (recency, lexical identity), also indicating that it has very limited if any understanding of CC.

**Table 7:** Accuracies for the semantic probe with our three calibration methods compared to no calibration.

		**Accuracies**				**Decision flips**			
**Model**	**Test sentence**	**−**	**S5**	**S6**	**S7**	**−**	**S5**	**S6**	**S7**
BERT_B_	S1	37.65	37.62	44.39	47.9	–	–	–	–
	S2	64.64	62.79	56.66	55.41	26.99	25.22	14.75	10.77
	S3	38.04	44.78	44.09	48.29	75.69	23.51	86.33	91.05
	S4	–	–	–	–	2.71	–	–	–
BERT_L_	S1	36.85	31.91	47.21	44.03	–	–	–	–
	S2	67.13	73.48	54.39	64.45	30.44	41.8	13.37	22.24
	S3	36.46	43.43	47.79	44.36	73.31	25.94	88.65	85.97
	S4	–	–	–	–	2.32	–	–	–
RoBERTa_B_	S1	61.6	58.76	42.13	62.32	–	–	–	–
	S2	52.85	51.35	71.33	60.25	9.92	8.67	31.13	10.86
	S3	62.21	55.17	43.04	62.76	76.19	22.04	79.03	74.75
	S4	–	–	–	–	2.76	–	–	–
RoBERTa_L_	S1	55.72	58.37	65.08	69.53	–	–	–	–
	S2	68.01	74.53	62.73	77.76	14.34	17.82	15.94	15.86
	S3	55.36	52.02	65.28	69.23	79.48	43.64	79.75	78.32
	S4	–	–	–	–	3.25	–	–	–
DeBERTa_B_	S1	41.61	36.41	32.79	43.27	–	–	–	–
	S2	42.95	43.04	33.77	42.36	24.21	24.4	8.79	7.49
	S3	41.92	38.64	32.39	43.31	74.58	17.83	72.29	64.42
	S4	–	–	–	–	1.67	–	–	–
DeBERTa_L_	S1	58.5	60.34	45.17	65.42	–	–	–	–
	S2	64.56	66.43	49.99	62.77	13.47	14.27	14.43	13.15
	S3	58.8	59.84	45.41	65.45	78.25	30.36	75.61	70.21
	S4	–	–	–	–	2.65	–	–	–
DeBERTa_XL_	S1	67.24	74.59	57.33	76.64	–	–	–	–
	S2	76.31	78.92	63.75	78.41	18.02	18.79	17.37	16.48
	S3	67.28	74.35	57.51	76.69	82.35	43.29	78.43	72.99
	S4	–	–	–	–	3.34	–	–	–

We report the average accuracy on the more difficult sentences in terms of recency bias (S1), the easier ones (S2), and vocabulary bias (S3), as well as the percentage of decisions flipped by changing from the base S1 to each sentence. Our calibration techniques are short (S5), name (S6), and adjective (S7).

Looking at the accuracies, we see that RoBERTa's and DeBERTa's scores are close to 50 (i.e., chance) accuracy for both S1 and S2. BERT models differ considerably as they seem to suffer from bias related to the order of the two CCs, but we can see that the average between them is also very close to chance. When we further look at the decision flips for each of the biases, we find that there is next to no bias related to the choice of names (S4). However, we can see a large bias related to both the recency of the correct answer (S2) and the choice of adjectives (S3). The recency bias is strongest in the BERT models, which also accounts for the difference in accuracies. For RoBERTa and DeBERTa models, the recency bias is small, but clearly present. In contrast, they exhibit far greater bias toward the choice of adjective, even going as far as 99.66% of decisions flipped by changing the adjective for DeBERTa base. This suggests that these models' decisions about which adjective to assign a higher probability is almost completely influenced by the choice of adjective, not the presence of the CC. Overall, we conclude that without calibration, all models seem to be highly susceptible to different combinations of bias, which completely obfuscate any underlying knowledge of the CC, leading to an accuracy at chance level across the board.

We therefore turn to our calibration methods, evaluating them first on their influence on the decision flip scores, which directly show if we were able to reduce the impact of the different types of bias. We report these only for order and vocabulary bias as we found name bias to be inconsequential. We report the complete results in Table 7. We see that across all models, while all three calibration methods work to reduce some bias, none does so consistently across all models or types of bias. Even in cases where calibration has clearly reduced the decision flip score, we find that the final calibrated accuracy is still close to 50%. This indicates that despite the effort to retrieve any knowledge that the models have about the CC, they are unable to perform clearly above chance, and we have therefore found no evidence that the investigated models understand and can use the semantics of the CC.

To investigate if this was result was exclusive to smaller, masked language models, we repeat our experiment and turn to larger, autoregressive models, more specifically, different sizes of OPT (Zhang et al., 2022).

### 6.3. Experiments on autoregressive language models

#### 6.3.1. Methods

##### 6.3.1.1. Probing setup

Since we concluded from our experiments with masked language models that none of them have reached significant performance on our task, we move on to investigating newer autoregressive models. We hope that as these models have been shown to perform significantly better on natural language understanding (NLU; Zhang et al., 2022), which is a prerequisite for our probing setup, their performance will be more directly indicative of their understanding of the CC in context.

As we can no longer perform our experiments on the basis of comparing the predictions for a given MASK token, we modify the setup such that our metric is based on the comparison of the perplexity of two competing whole sentences. Our main idea is to no longer work with antonyms but instead create contrast by swapping the two names in the last sentence. Given the context “The ADJ1-er you are, the ADJ2-er you are. NAME1 is ADJ1-er than NAME2.,” we contrast the perplexities of “Therefore, NAME1 will be ADJ2-er than NAME2” and “Therefore, NAME2 will be ADJ2-er than NAME1.” While the sentences are bag-of-words equivalent, only the first one follows from the context. This has the additional effect of removing the confounding factor of the second sentence with antonyms from the factors that influence the model's performance. For example, we would now contrast “The stronger you are, the faster you are. Terry is stronger than John. Therefore, Terry will be faster than John” with “The stronger you are, the faster you are. Terry is stronger than John. Therefore, John will be faster than Terry.”

##### 6.3.1.2. Name bias

Similarly to our previous experiment in Section 6.2.1, we hypothesize biases to this setup and test them. Our “adjective bias” and “recency bias” are not immediately applicable here, as we no longer have a masked token.

However, we expect that models might consistently prefer one final sentence, which is the one that changes the acceptability of the entire test phrase, over another, regardless of context. To test this, we construct a second pair of sentences, where the names are swapped both times. This means that when iterating through all 4-tuples of sentences that belong together, we can now compare all four and count only those as valid results where either both pairs were correctly classified or both were incorrectly classified. For the others, where one was correct and the other incorrect, this indicates that the model preferred one final sentence over the other in all contexts. We count how many times this occurs to quantify the strength of this name bias in a model.

#### 6.3.2. Initial results

For our results, we consider each four-tuple of sentences *S*_8_-*S*_11_. We perform perplexity comparisons twice: firstly, we expect the perplexity of *S*_8_ to be lower than that of *S*_9_; secondly, we anticipate the perplexity of *S*_10_ to be lower than that of *S*_11_. We denote *C* to represent the count of correct results where both conditions are met, *I* to represent the count of incorrect results where both conditions fail, and *In* to represent the count of inconclusive results where one condition is met and the other is not.

**Figure 2:**
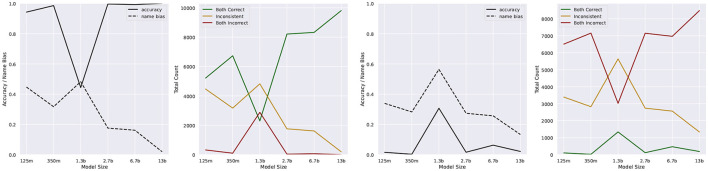
Accuracy and name bias scores for test sentences S8–S11 on the left and S12–S15 on the right, on different sizes of OPT.

The general trend for these three counts can be seen in the right half of Figure 2. As the models increase in size, *C* rises and *In* drops, with *I* remaining generally low. The only exception to this is the OPT-1.3b model, for unknown reasons.

We then develop two more abstract metrics based on these counts:

1. We define the accuracy, *A*, as the number of correct responses divided by the number of valid responses (correct and incorrect ones). In mathematical terms: $A=\frac{C}{C+I}$.
2. As a complementary metric, we define the “name bias,” *B*, as the percentage of inconclusive responses over total responses. Mathematically, $B=\frac{In}{C+I+In}$.

We use “name bias” to denote situations where the model consistently favored one of the two possibilities for the last sentence, indicating a possible bias for this sentence, perhaps due to the order of names and the combination with the particular adjective.

Our observations show that *A* remains consistently high (with the exception of 1.3 b) and *B* decreases as the model size increases.

These results were initially encouraging for the hypothesis that larger, autoregressive models are able to capture the semantics of the CC. However, there is one important possibility for bias in all four sentences: the correct answer is consistently that in which the two names are in the same order in both sentences. We therefore have to examine the possibility that the near-perfect accuracy displayed in our task is merely due to the name order being parallel and not to any deeper understanding of the sentences.

#### 6.3.3. Additional experiment

We therefore construct four additional sentences, named S12–S15 in Table 8. They are constructed with “less,” to ensure that the correct answer is now the one where the order of names is swapped. We rerun the same experiment as before with these sentences. We expect that if the model was merely preferring the parallel order of names, the accuracy would be close to zero, whereas a continued good accuracy would indicate that it formed a deeper understanding of the task.

**Table 8:** Overview of the schemata of test scenarios S8–S15, used for semantic probing for autoregressive language models.

**No**.	**Name order**	**Validity**	**Sentence schema**
S8	Same	True	The ADJ1-er you are, the ADJ2-er you are. NAME1 is ADJ1-er than NAME2.
			Therefore, NAME1 will be ADJ2-er than NAME2.
S9		False	The ADJ1-er you are, the ADJ2-er you are. NAME1 is ADJ1-er than NAME2.
			Therefore, NAME2 will be ADJ2-er than NAME1.
S10		True	The ADJ1-er you are, the ADJ2-er you are. NAME2 is ADJ1-er than NAME1.
			Therefore, NAME2 will be ADJ2-er than NAME1.
S11		False	The ADJ1-er you are, the ADJ2-er you are. NAME2 is ADJ1-er than NAME1.
			Therefore, NAME1 will be ADJ2-er than NAME2.
S12	Flipped	True	The ADJ1-er you are, the ADJ2-er you are. NAME1 is less ADJ1 than NAME2.
			Therefore, NAME2 will be ADJ2-er than NAME1.
S13		False	The ADJ1-er you are, the ADJ2-er you are. NAME1 is less ADJ1 than NAME2.
			Therefore, NAME1 will be ADJ2-er than NAME2.
S14		True	The ADJ1-er you are, the ADJ2-er you are. NAME2 is less ADJ1 than NAME1.
			Therefore, NAME1 will be ADJ2-er than NAME2.
S15		False	The ADJ1-er you are, the ADJ2-er you are. NAME2 is less ADJ1 than NAME1.
			Therefore, NAME2 will be ADJ2-er than NAME1.

The results in Figure 2 show that unfortunately the former was the case: all values are approximately inverted compared to the first experiment. If the model had formed an understanding of the CC in this task, our reformulation of the task could not have completely destroyed the performance. We therefore conclude that none of the models, at least in this setup, have demonstrated an understanding of the CC.

### 6.4. Problem analysis

Different conclusions might be drawn as to why none of these models have learned the semantics of the CC. Firstly, they might not have seen enough examples of it to have formed a general understanding. Given the amount of examples that we were able to find in C4, and the overall positive results from the syntax section, we find this to be unlikely. Secondly, it could be argued that models have never had a chance to learn what the CC means because they have never seen it together with a context in which it was immediately applied, and do not have the same opportunities as humans available, which would be to either interact with the speaker to clarify the meaning, or to make deductions using observations in the real world. This is in line with other considerations about large PLMs acquiring advanced semantics, even though it has for many phenomena been shown that pre-training is enough (Radford et al., 2019). Lastly, it might be possible that the type of meaning representation required to solve this task is beyond the current transformer-style architectures. Overall, our finding that PLMs do not learn the semantics of the CC adds to the growing body of evidence that complex semantics like negation (Kassner and Schütze, 2020) is still beyond state-of-the-art PLMs.

## 7. Conclusion

We have made a first step toward a thorough investigation of the compatibility of the paradigm of CxG and the syntactic and semantic capabilities exhibited by state-of-the-art large PLMs. For this, we chose the English comparative correlative, one of the most well-studied constructions, and investigated if large PLMs based on masked language modeling have learned it, both syntactically and semantically. We found that even though they are able to classify sentences as instances of the construction even in difficult circumstances, they do not seem to be able to extract the meaning it conveys and use it in context, indicating that while the syntactic aspect of the CC is captured in pretraining of these models, the semantic aspect is not. We then repeated a modified version of our semantic experiments with larger, autoregressive language models, and found that they were similarly unable to capture the semantics of the construction.

## 8. Limitations

As our experimental setup requires significant customization with regards to the properties of the specific construction we investigate, we are unable to consider other constructions or other languages in this work. We hope to be able to extend our experiments in this direction in the future. Our analysis is also limited—as all probing papers are—by the necessary indirectness of the probing tasks: we cannot directly assess the model's internal representation of the CC, but only construct tasks that might show it but are imperfect and potentially affected by external factors.

## Data availability statement

The datasets presented in this study can be found in online repositories. The names of the repository/repositories and accession number(s) can be found at: https://github.com/LeonieWeissweiler/ComparativeCorrelative.

## Author contributions

LW designed the original research question together with VH. They wrote and discussed the linguistics background as well as the motivation for the experiments and the specifics of the syntax experiments. The specifics of the semantics experiments were designed by LW and AK. HS acted as main advisor to LW, VH, and AK and gave guidance on the process throughout. All authors contributed to the article and approved the submitted version.

## Footnotes

^1^ https://www.deepl.com/translator

^2^ http://neural-nets-for-cognition.net

^3^Note that an alternative reading of this sentence exists: the numeral “two” forms the noun phrase by itself and “The harder” is still interpreted as part of the CC. The sentence is actually a positive instance on this interpretation. We regard this reading as very improbable.
