# Supplementary material for: Explaining pretrained language models' understanding of linguistic structures using construction grammar
Source: Front Artif Intell. 2023 Oct 12;6:1225791. doi: 10.3389/frai.2023.1225791 (PMC10600487; doi:10.3389/frai.2023.1225791)
Supplement: Supplementary file 1 [file Data_Sheet_1.pdf]

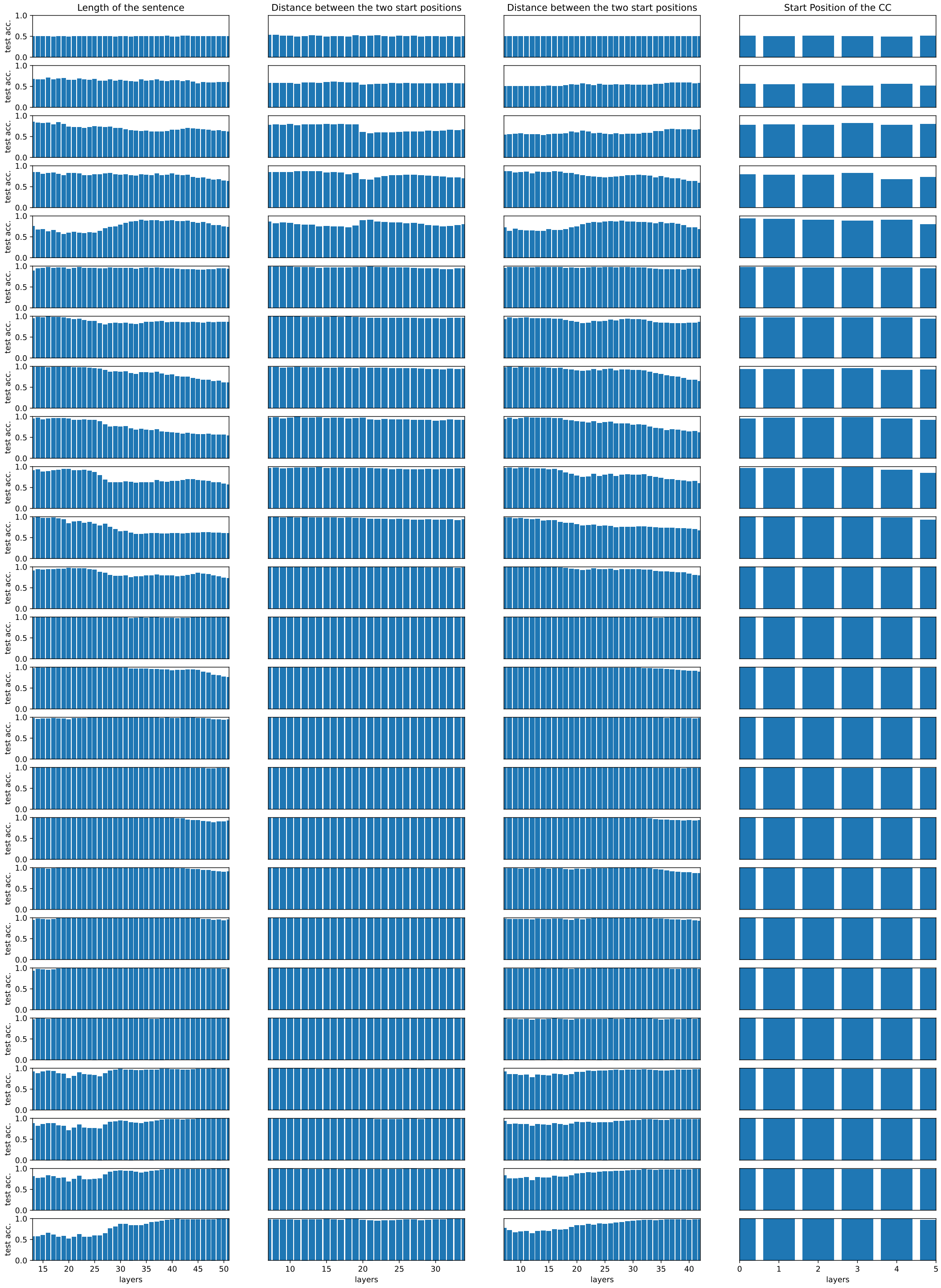

Figure A1: Full results for $BERT_{LARGE}$ on artificial data. Columns indicate the variable that the training and test set controls for.

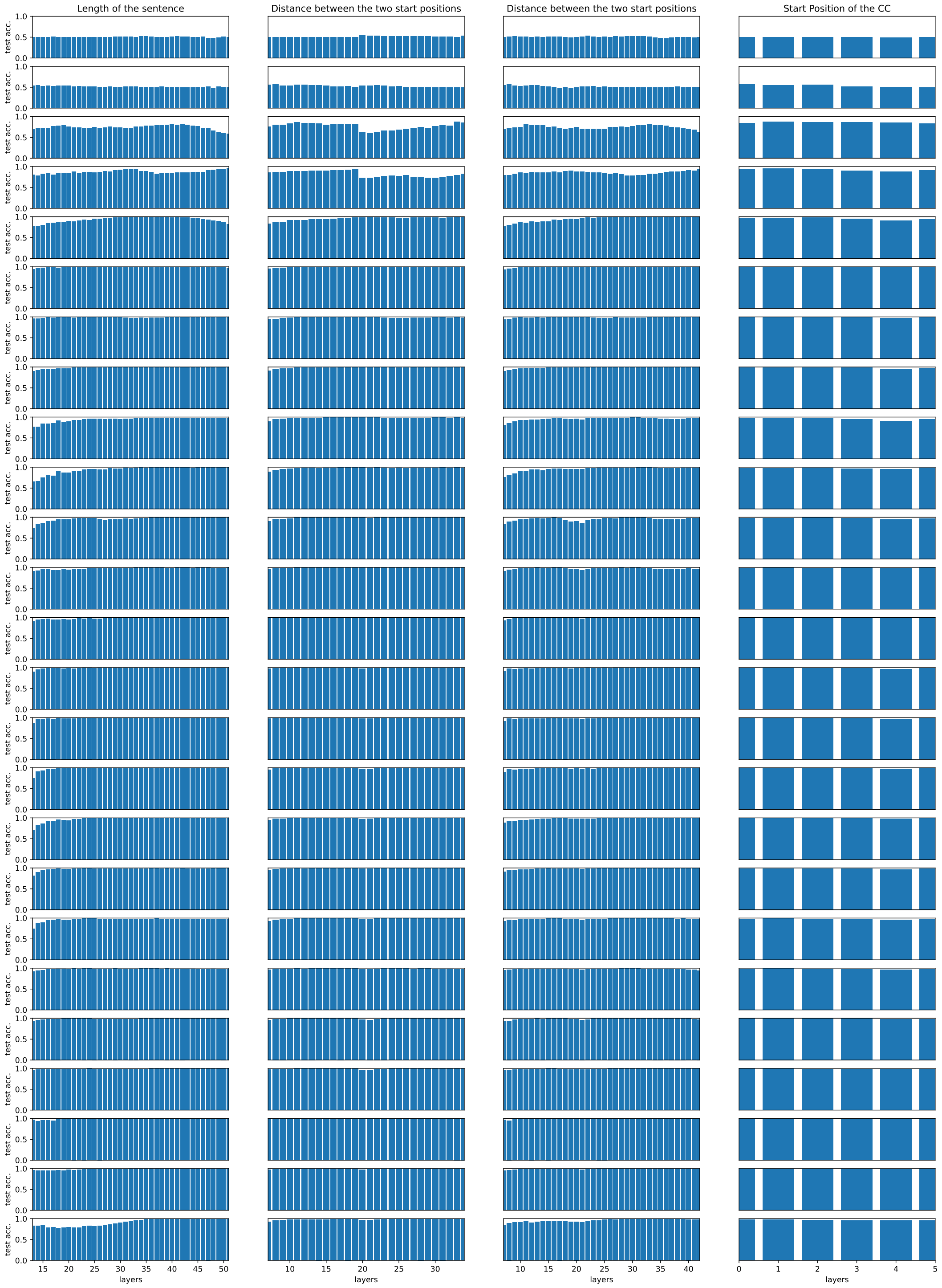

Figure A2: Full results for $\text{RoBERTa}_{\text{LARGE}}$ on artificial data. Columns indicate the variable that the training and test set controls for.

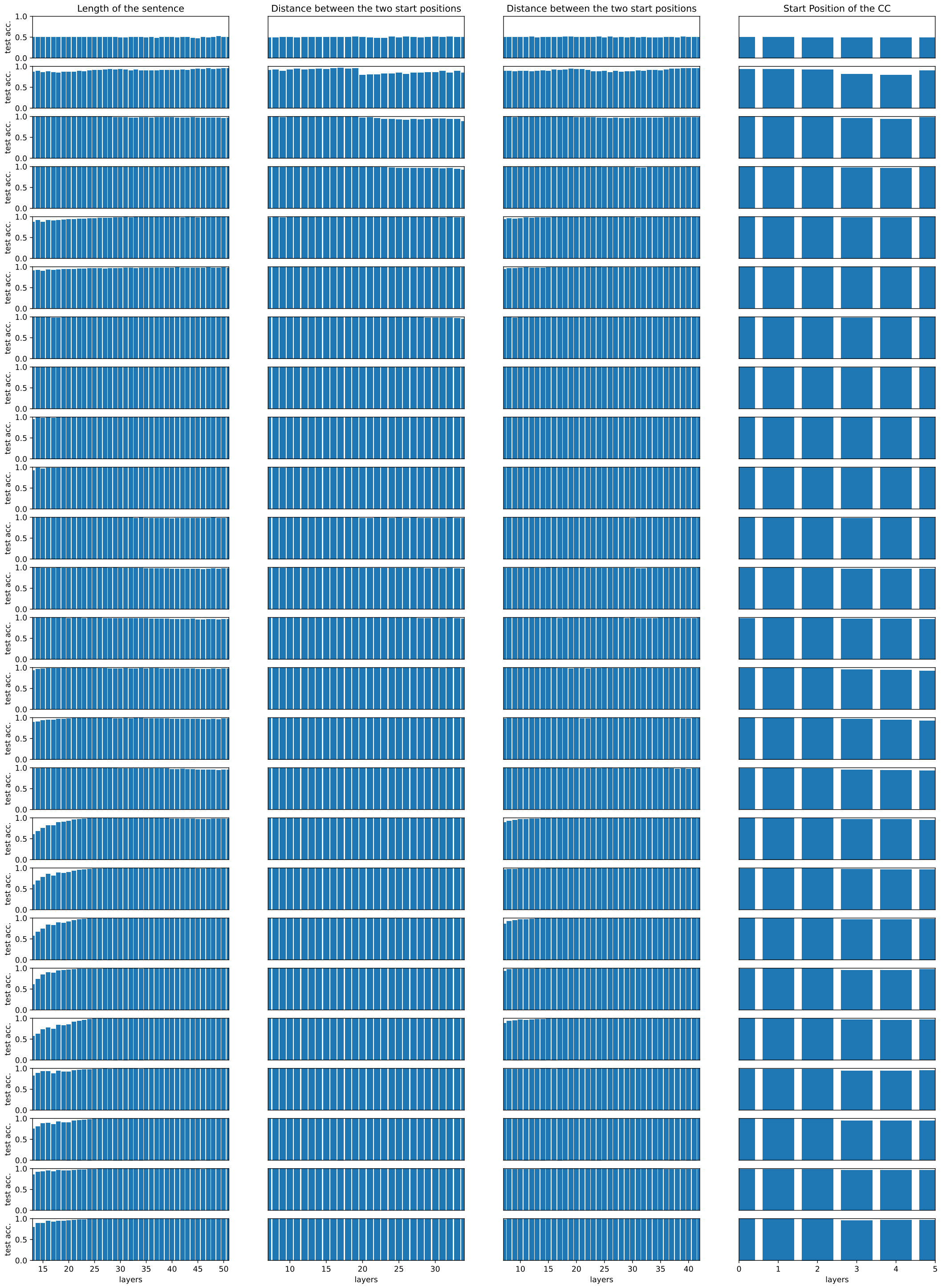

Figure A3: Full results for DeBERTa$_{\mathrm{LARGE}}$ on artificial data. Columns indicate the variable that the training and test set controls for.

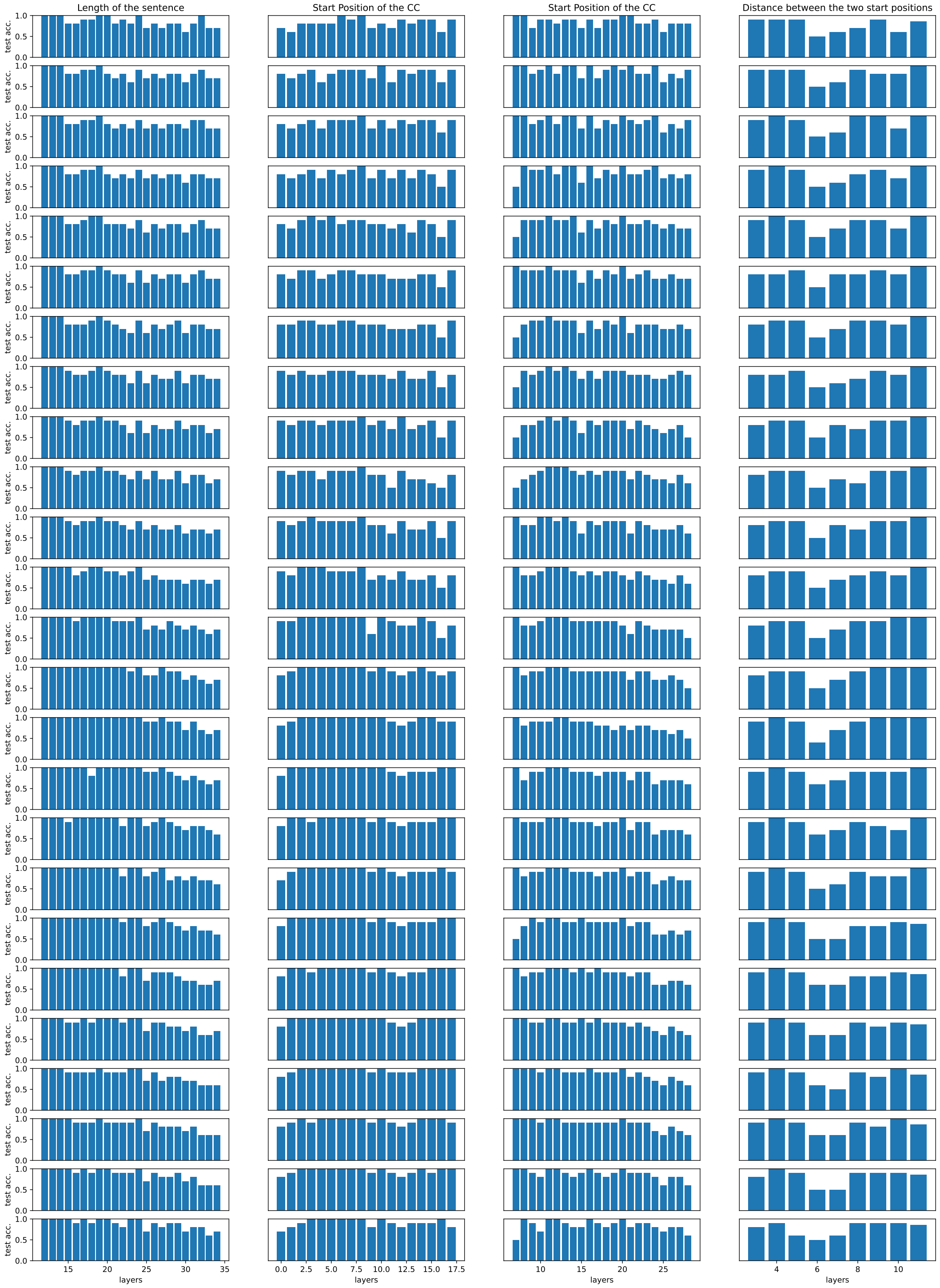

Figure A4: Full results for $\text{BERT}_{\text{LARGE}}$ on corpus data. Columns indicate the variable that the training and test set controls for.

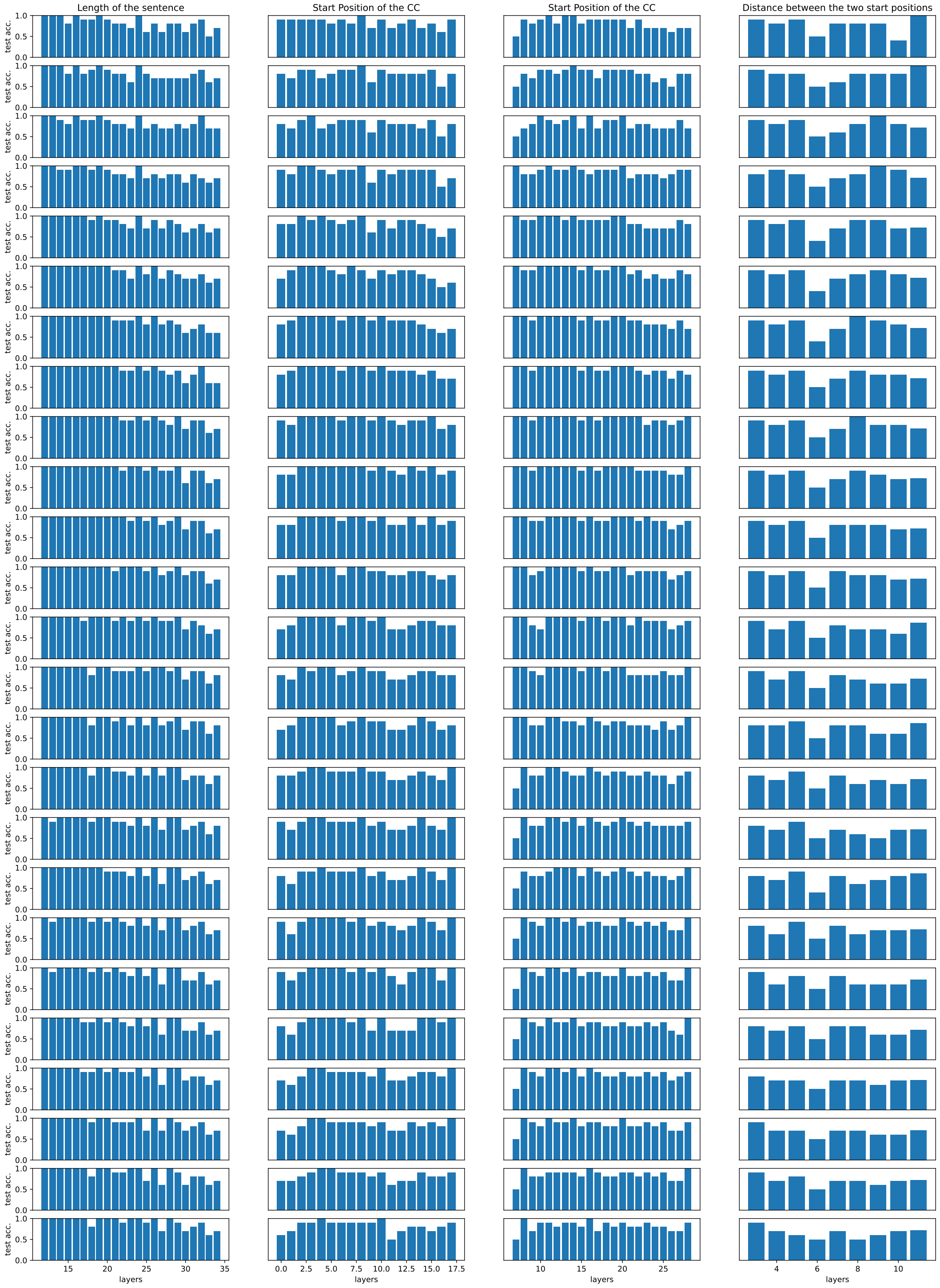

Figure A5: Full results for $\text{RoBERTa}_{\text{LARGE}}$ on corpus data. Columns indicate the variable that the training and test set controls for.

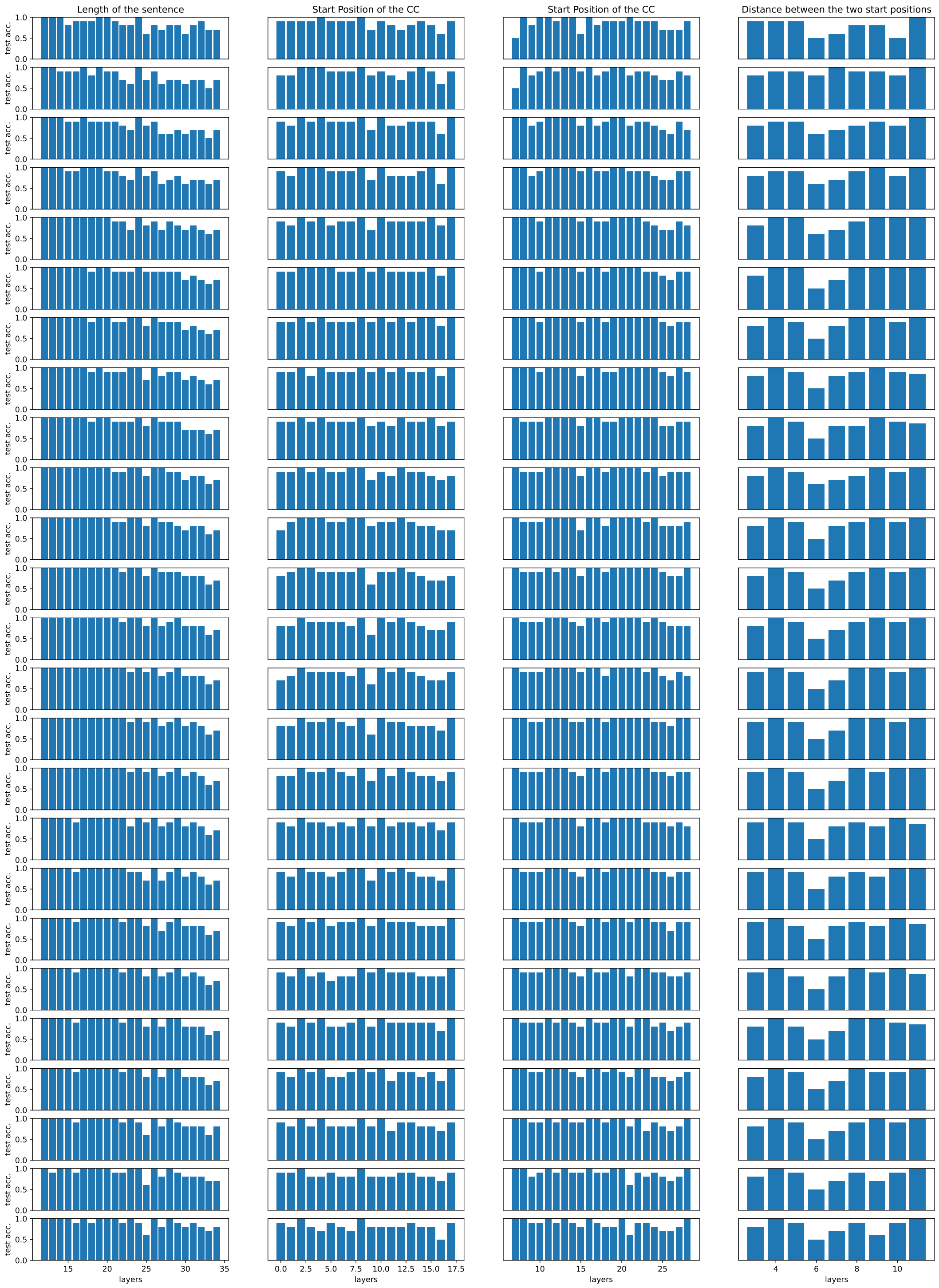

Figure A6: Full results for DeBERTa$_{\text{LARGE}}$ on corpus data. Columns indicate the variable that the training and test set controls for.
